# RAS Mutations in Advanced Colorectal Cancer: Mechanisms, Clinical Implications, and Novel Therapeutic Approaches

**DOI:** 10.3390/medicina61071202

**Published:** 2025-06-30

**Authors:** Osman Sütcüoğlu, Hasan Çağrı Yıldırım, Elvina Almuradova, Damla Günenç, Şuayib Yalçın

**Affiliations:** 1 Department of Medical Oncology, Gazi University, Ankara 06560, Turkey; 2 Department of Medical Oncology, Niğde Research and Training Hospital, Niğde 51100, Turkey; hasan-cagri@windowslive.com; 3 Department of Medical Oncology, Medicana International İzmir Hospital, İzmir 35170, Turkey; almuradovaelvina@gmail.com; 4 Department of Medical Oncology, Ege University, İzmir 35040, Turkey; damla.gunenc@ege.edu.tr; 5 Department of Medical Oncology, Hacettepe University, Ankara 06230, Turkey; suayibyalcin@yahoo.com

**Keywords:** colon cancer, RAS mutation, molecular oncology, survival

## Abstract

Colorectal cancer (CRC) remains a leading cause of cancer-related mortality globally, posing significant treatment challenges, particularly in its metastatic form (mCRC). This review comprehensively examines the pivotal role of RAS mutations, specifically KRAS and NRAS, which are detected in approximately 40–45% of mCRC cases, and their impact on treatment decisions and patient outcomes. We assess the effectiveness of standard treatments within the RAS mutant population, highlighting the challenges and limitations these therapies face. Recent advancements in targeted therapies, particularly the focus on novel agents such as KRAS G12C inhibitors, including sotorasib and adagrasib, have shown promising efficacy in overcoming resistance to conventional treatments. Furthermore, this review discusses future directions, emphasizing the need for research into non-RAS targets to address the complexities of resistance mechanisms and improve therapeutic outcomes. This review aims to provide a detailed overview of the current treatments and innovative approaches, supporting the development of personalized management strategies for patients with mCRC.

## 1. Introduction

Colorectal cancer (CRC) continues to be one of the leading causes of cancer-related deaths worldwide. The treatment of metastatic colorectal cancer (mCRC) is complex, influenced by numerous clinical and molecular factors. It is known that RAS mutations, which are one of the first molecular parameters examined in patients diagnosed with mCRC, play a critical role in the selection of treatment and prognosis of the disease. These mutations, predominantly found in the KRAS and NRAS genes, are detected in approximately 40–45% of mCRC, and the correct management of patients with these mutations will directly change the prognosis of mCRC [1].

This review focuses on the effects of RAS mutations on mCRC. We aim to examine the impact of these mutations on treatment outcomes, their roles as predictive and prognostic biomarkers, and the evolving landscape of therapeutic strategies tailored to RAS mutation status. Recent clinical trials targeting KRAS mutations have shown promising developments (especially K-ras 12c inhibitors sotorasib and adagrasib) that herald a potential shift in treatment options [2,3]. To support this review, we describe our methodology for literature selection, including databases searched (PubMed, Scopus, and ClinicalTrials.gov), keywords used (e.g., “RAS mutation”, “colorectal cancer”, “KRAS”, and “targeted therapy”), and the timeframe (January 2010 to March 2025). Both peer-reviewed original articles and clinical trial results were considered. This approach improves the transparency and reproducibility of our review. This review aims to synthesize the current therapeutic landscape and emerging treatments in metastatic colorectal cancer with RAS mutations, highlighting both direct and indirect targeting strategies and discussing their translational relevance for clinical practice.

### 1.1. RAS Mutations: Function and Impact on Treatment Resistance

#### 1.1.1. RAS Function in Colorectal Carcinoma

**Figure 1:**
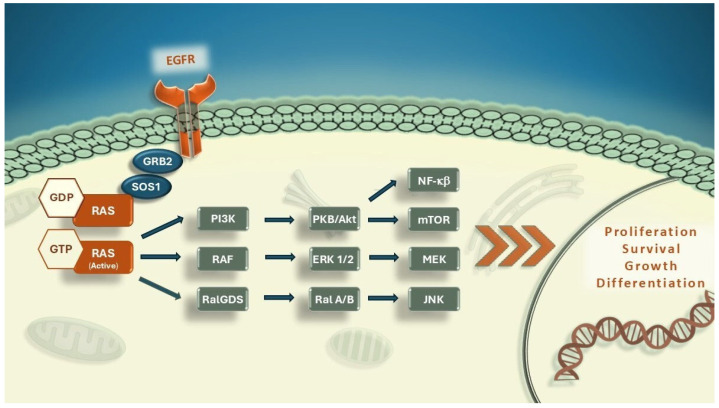
RAS-effector signaling pathways. PKB/Akt, Protein kinase B; ERK, Extracellular signal-regulated kinase; GDP, guanosine diphosphate; GRB2, Growth factor receptor bound protein 2; GTP, Guanosine triphosphate; EGFR, Epithelial growth factor receptor; JNK, c-Jun N-terminal kinases; MEK, Mitogen-activated protein kinase; mTOR, Mammalian target of rapamycin, NF-κB, Nuclear factor-κB; PI3K, Posphatidylinositol 3-kinase; RAF, RAF proto-oncogene serine/threonine-protein kinase; RalGDS, Ral guanine nucleotide dissociation stimulator; RAS: Rat sarcoma; SOS1, Son of sevenless homolog-1.

RAS proteins function as binary molecular switches that control intracellular signaling networks and regulate proliferation, differentiation, and survival functions (Figure 1) [4].

In colorectal cancer, mutations in the RAS gene family (mostly KRAS and NRAS) lead to the synthesis of actively functioning RAS proteins. These mutations lock RAS proteins in an active GTP-bound state, constitutively activating pathways such as MAPK and PI3K, independent of external growth signals [4]. This continuous signaling facilitates dysregulated cell proliferation and survival and underscores the aggressive features of mCRC.

In colorectal epithelial cells, oncogenic mutations in KRAS or NRAS—most commonly affecting codons 12, 13, or 61—result in the constitutive activation of RAS, maintaining it in a GTP-bound state independent of upstream EGFR stimulation [5]. This aberrant activation leads to persistent MAPK and PI3K/AKT signaling, driving uncontrolled cell proliferation and survival, thereby contributing to colorectal tumorigenesis [5]. Beyond their effect on signaling, RAS mutations in CRC have been implicated in remodeling the tumor microenvironment, promoting angiogenesis, modulating immune infiltration, and enhancing metastatic potential [6]. They are also associated with lower response rates to chemotherapy and shorter overall survival in several clinical cohorts [7].

In summary, the functional consequences of KRAS and NRAS mutations in colorectal cancer are multifaceted, influencing not only intracellular signal transduction and therapeutic response but also tumor progression and systemic disease behavior. These insights form the rationale for ongoing efforts to develop targeted therapies that can bypass or counteract aberrant RAS signaling in mCRC.

#### 1.1.2. The Function of RAS Mutations in Anti-Cancer Therapy Resistance

RAS mutations significantly contribute to resistance to EGFR-targeted therapies, such as cetuximab and panitumumab, commonly used in the treatment of mCRC [8]. Typically, RAS activation facilitates EGFR signaling. When RAS remains mutated and persistently active, EGFR inhibition has minimal effects on downstream signaling, thereby rendering these therapies ineffective. This mechanism is crucial in assessing patient eligibility for EGFR-targeted therapies, as only individuals with wild-type RAS genes are expected to benefit, highlighting the predictive importance of RAS mutation status.

#### 1.1.3. Resistance Mechanisms and Evolution of Targeted Therapies in RAS-Mutant Colorectal Cancer

Despite the effectiveness of chemotherapy and anti-VEGF treatments, the presence of RAS mutations may cause treatment resistance due to their effects on the tumor microenvironment and the variability in response among patients. The development of direct RAS inhibitors offers promising new developments to improve treatment efficacy. The development of KRAS G12C inhibitors and their use with anti-EGFR drugs has shown promise in clinical trials, especially in second and subsequent lines of treatment [2,3]. In addition, therapeutic alternatives are being developed for patients with other specific RAS mutations. These developments emphasize the importance of personalized treatment regimens according to the molecular or genetic profile and different molecular characteristics of each tumor.

### 1.2. Prognostic and Predictive Role of RAS Mutation in Patients Diagnosed with mCRC

#### 1.2.1. First-Line Therapy: Anti-EGFR and Anti-VEGF Trials

Approximately 45% of patients diagnosed with mCRC have KRAS mutations, and the presence of mutations is important both in terms of prognosis [9,10]. Therefore, molecular analysis should be performed before making a treatment decision in patients diagnosed with mCRC.

Studies indicate that patients with KRAS mutations diagnosed with mCRC not only exhibit a poorer prognosis but also respond inadequately to anti-EGFR therapies [10]. Lievre et al. assessed the effectiveness of a cetuximab–irinotecan combination in patients who had progressed following chemotherapy, revealing that all 11 patients responding to treatment were KRAS WT, while 13 out of 19 non-responders had KRAS mutations [11]. A comparison of cetuximab and the best supportive care in 394 mCRC patients who had undergone multiple treatment lines did not demonstrate cetuximab’s superiority in those with KRAS-activating mutations [12]. In the COIN study, the effectiveness of cetuximab added to chemotherapy was evaluated, and in contrast to expectations, no positive or detrimental effect of the addition of anti-EGFR was observed in RAS-wild and RAS-mutated patients [13]. The PRIME study, which evaluated the efficacy of adding panitumumab to FOLFOX4 treatment in mCRC patients, found that the KRAS WT group benefited in terms of mPFS and mOS. In contrast, a detrimental effect was observed in the KRAS exon 2 mutant group [14]. Further analysis of molecular subgroups in this study showed that adding panitumumab significantly improved mPFS and mOS in the non-mutated KRAS group but was ineffective in patients with KRAS non-exon 2 mutations [15]. However, studies have also shown that the unresponsiveness to anti-EGFR therapy varies with the presence of the KRAS G13D mutation. The median PFS and mOS were significantly better in KRAS G13D mutant patients resistant to chemotherapy than those with other KRAS mutations [16]. Despite this, when treated with cetuximab, the KRAS G13D mutant group had a similar mPFS and mOS to the KRAS WT group. However, the objective response rate was low, suggesting that combining cetuximab with chemotherapy might be more effective than cetuximab alone.

In studies evaluating the addition of bevacizumab to chemotherapy agents, the addition of bevacizumab contributes to PFS, regardless of KRAS mutation status [17,18]. The PACCE study evaluated the addition of panitumumab to chemotherapy and bevacizumab, showing that the combined use of two biological agents may result in adverse outcomes irrespective of RAS mutation status [19]. In the TRIBE trial comparing the effectiveness of triplet plus bevacizumab treatment and doublet plus bevacizumab treatment, its contribution to survival was demonstrated, and it was shown to be effective regardless of RAS mutation status [20]. In this study, the survival time of RAS mutant patients was poorer than that of RAS-BRAF wild patients but was better than that of BRAF mutant patients. From all of these studies, we can conclude that the effectiveness of anti-EGFR agents is poor, and bevacizumab is effective in RAS mutant patients.

Among the pivotal trials evaluating targeted therapies in mCRC, the FIRE-3 and CALGB/SWOG 80405 studies offer important insights regarding the impact of RAS mutation status on treatment efficacy. In the FIRE-3 trial, the addition of cetuximab to FOLFIRI demonstrated improved overall survival in RAS wild-type patients, whereas no benefit was observed in those with RAS mutations. [8]. Similarly, the CALGB/SWOG 80405 trial showed that both cetuximab and bevacizumab provided comparable outcomes when combined with chemotherapy in the overall population, but the benefit of anti-EGFR therapy was again limited to RAS wild-type tumors [21]. These findings underscore the importance of comprehensive RAS genotyping in guiding first-line biologic selection in metastatic colorectal cancer. Phase III randomized clinical studies conducted in first-line colorectal cancer and detailed results of these studies according to RAS mutation status are presented in Table 1.

As these phase 3 randomized controlled studies indicate, anti-EGFR agents should not be used as first-line treatment in KRAS mutant tumors, and extended RAS analysis should be conducted before treatment initiation.

In addition to RAS activation, HER2 overexpression/amplification contributes to resistance against anti-EGFR therapies that target the epidermal growth factor receptor pathway, a key player in CRC tumorigenesis. HER2 overexpression/amplification occurs in 3–5% of patients with mCRC, rising to 5–14% among those with RAS WT [25]. As the use of anti-HER2 therapies in the treatment of mCRC advances, emerging data suggest a differential effect of these therapies based on RAS mutation status. In the phase 2 MyPathway study, which focused on HER2-positive patients with mCRC, mPFS and mOS were poorer for patients with RAS mutations treated with trastuzumab-pertuzumab compared to the RAS WT group [26]. Furthermore, RAS mutant patients did not achieve any treatment response with trastuzumab-pertuzumab. Notably, RAS mutant patients were excluded from the MOUNTAINEER study, which evaluated the efficacy of tucatinib, and only two RAS mutant patients were included in the DESTINY-CRC01 study, assessing the efficacy of trastuzumab deruxtecan [27,28]. Given the minimal or absent inclusion of RAS mutant patients in these studies and the observed lack of treatment response in the MyPathway study, it is evident that the effectiveness of HER2-targeted therapies is significantly limited in the presence of a KRAS mutation in tumors that are HER2-amplified and/or overexpressed.

These data strongly support the use of anti-EGFR agents exclusively in RAS wild-type tumors and reinforce the need for extended RAS testing prior to initiating targeted therapies in mCRC.

#### 1.2.2. Second-Line Therapy: Angiogenesis Inhibitors

Current data from clinical trials suggests potential advantages for individuals with mCRC by maintaining angiogenesis inhibition even after initial progression [29]. Evidence from randomized phase III studies suggests that the sustained inhibition of tumor angiogenesis using agents like bevacizumab, aflibercept, or ramucirumab beyond the initial progression may contribute to enhanced OS [30].

##### Bevacizumab

In the ECOG 3200 study, the addition of bevacizumab to chemotherapy, specifically FOLFOX 4 and chemotherapy alone, demonstrated significantly prolonged mOS and mPFS compared to the control groups [31,32]. In the phase III ML18147 trial, adding bevacizumab improved mPFS in both KRAS WT and mutant tumors. mOS was prolonged in KRAS WT tumors with bevacizumab plus chemotherapy, while similar outcomes were observed in mutant KRAS tumors [32]. The interaction test by KRAS status showed no statistically significant difference in bevacizumab efficacy between KRAS mutant and wild-type tumors, with *p* = 0.443 for PFS and *p* = 0.126 for OS [23]. The continuation of bevacizumab beyond the first-line triplet chemotherapy/bevacizumab regimen was evaluated in the TRIBE-2 trial [33]. The results showed a significant advantage for upfront FOLFOXIRI plus bevacizumab in terms of PFS2 with an mPFS2 of 19.1 months vs. 16.4 months for the comparator arm (HR 0.74, *p* < 0.001).

##### Aflibercept

The phase III VELOUR trial evaluates the efficacy of FOLFIRI plus aflibercept beyond progression on oxaliplatin-based first-line treatment [34]. FOLFIRI, in combination with aflibercept, not only significantly extended mOS when contrasted with FOLFIRI plus placebo but also exhibited prolonged mPFS and a nearly doubled response rate. In biomarker analysis, HRs for mOS favored WT KRAS exon 2 and extended RAS, although no significant interaction was found (*p* = 0.38) despite a trend favoring RAS WT [35].

##### Ramucirumab

Ramucirumab is considered a viable option for second-line therapy in mCRC patients who have experienced progression after initial treatment with bevacizumab and chemotherapy [36]. The RAISE trial aimed to assess the effectiveness of ramucirumab in combination with FOLFIRI compared to placebo plus FOLFIRI in mCRC cases [37]. The results showed that mOS and mPFS were significantly better in the group receiving ramucirumab. The KRAS exon 2 mutation status served as a stratification factor, with approximately half of the patients having mutant KRAS tumors. Ramucirumab demonstrated directional improvements in mOS and mPFS compared to placebo in patients with mutant KRAS tumors. Notably, improvements in overall survival were observed with ramucirumab across all patients, including those with RAS/BRAF mutations, with a significant improvement in mPFS observed in the RAS mutant patients (*p* = 0.021) [36].

### 1.3. Differential Benefits of Single-Agent Bevacizumab Maintenance Therapy: Subgroup Analysis Insights

In the AIO-KRK-0207 study, subgroup analysis for mPFS demonstrated a significant advantage in patients with wild-type tumors who received bevacizumab maintenance therapy compared to those who did not (HR 2.21, 95% CI 1.38–3.52) [38]. However, no significant difference was observed in patients with RAS or BRAF mutations (HR 1.19, 95% CI 0.84–1.70). In the PRODIGE-9 study, subgroup assessment for mPFS indicated a favorable HR of bevacizumab as a single agent in KRAS WT patients compared to those receiving no maintenance: 0.72 (95% CI 0.54–0.95), but this was not significant in KRAS mutation patients (HR: 1.07, 95% CI 0.79–1.44) [39]. Moreover, a meta-analysis by Lisa Salvatore et al. revealed consistent benefits with bevacizumab across clinical subgroups, except for patients with RAS mutations [40]. Exploratory analysis showed a greater benefit from bevacizumab as a single agent in RAS WT patients (HR 0.56, 95% CI: 0.37–0.83) compared to patients with RAS mutations (HR 0.91, 95% CI: 0.70–1.18), indicating a significant interaction between maintenance therapy and RAS status (*p* = 0.048). In a meta-analysis consisting of 14 phase III trials, the efficacy analysis aimed to compare outcomes between patients with wild and mutant tumors. The study demonstrated that the improvement in OS was significantly more pronounced in patients with RAS WT tumors than those with RAS mutant tumors across all included studies (*p* for interaction = 0.003) [41]. Furthermore, the addition of anti-angiogenic treatment was significantly less effective in RAS mutant tumors compared to wild-type tumors, with a notable interaction effect (wt: HR = 0.78 vs. mut: HR = 0.91) (Table 2).

Unlike anti-EGFR therapies, anti-angiogenic agents such as bevacizumab, aflibercept, and ramucirumab retain efficacy in RAS-mutant tumors and remain valuable components of both first- and second-line treatment strategies in this population.

### 1.4. Third-Line and Beyond: KRAS G12C Inhibitors and Novel Agents

#### 1.4.1. Regorafenib

Regorafenib was introduced into clinical practice in two pivotal studies for the treatment of metastatic colorectal cancer [42,43]. The CORRECT study, which evaluated 760 patients (255 placebo vs. 505 regorafenib), demonstrated the superiority of regorafenib in the primary endpoint of overall survival at 6.4 months vs. 5.0 months (HR) 0.77 (95% CI 0.64–0.94 *p* = 0.0052) [42]. The international phase 3 CONCUR study evaluated regorafenib in a larger population of Asian patients with refractory mCRC [43]. The median OS in the regorafenib group was significantly better at 8.8 months compared to 6.3 months in the placebo group (HR) 0.55 (95% CI 0.40–0.77). When assessing efficacy concerning RAS mutation status in the CORRECT trial, the overall survival hazard ratio (HR) for regorafenib vs. placebo was 0.87 (95% CI, 0.67–1.12) in the RAS mutant group and 0.65 (95% CI, 0.48–0.90) in the RAS wild-type group. Similarly, in the CONCUR trial, the overall survival HR for regorafenib vs. placebo was 0.65 (95% CI, 0.36–1.15) in the RAS mutant group and 0.59 (95% CI, 0.34–1.01) in the RAS wild-type group.

#### 1.4.2. TAS 102

The phase 3 RECOURCE trial evaluated TAS-102 vs. placebo in metastatic CRC patients refractory to standard chemotherapies [44]. TAS-102 significantly improved mOS to 7.1 months compared to 5.3 months with placebo (HR for death: 0.68, 95% CI 0.58–0.81). Additionally, TAS-102 prolonged the time to worsening performance status compared to placebo, emphasizing its efficacy in delaying disease progression [44]. A comprehensive meta-analysis showed that TAS-102 demonstrated significant mOS benefits in both KRAS WT patients (HR: 0.66, 95% CI 0.55, 0.79) and KRAS mutant patients (HR: 0.75, 95% CI 0.62, 0.91) [45].

### 1.5. Trifluridine/Tipiracil Plus Bevacizumab

The multinational Phase III SUNLIGHT study assessed FTD/TPI plus bevacizumab as third-line therapy for patients with unresectable metastatic CRC [46]. The addition of bevacizumab to FTD/TPI showed a mOS of 10.8 months compared to 7.5 months with FTD/TPI alone, marking a 39% reduction in the risk of death. Moreover, mPFS in the investigational arm reached 5.6 months compared to 2.4 months in the control arm, resulting in a 56% reduction in disease progression or death risk. The post hoc analysis of the Phase III SUNLIGHT trial demonstrated that FTD/TPI plus bevacizumab treatment resulted in better overall survival compared to FTD/TPI alone in both the RAS mutant and RAS wild-type groups; the median OS (mOS) was 10.1 months vs. 7.1 months for mRAS and 11.4 months vs. 7.4 months for wtRAS, respectively [47].

#### Frequintinib

Fruquintinib is an oral agent that inhibits VEGFR-1, 2, and 3. Its efficacy in patients with disease progression after standard treatments has been demonstrated in the phase III FRESCO-1 study in China and the international FRESCO-2 trial [48,49]. In FRESCO-1, fruquintinib showed significant benefits in both overall survival (OS) and progression-free survival (PFS) compared to placebo. Subgroup analyses reported an OS hazard ratio (HR) of 0.56 in KRAS wild-type (WT) patients and 0.75 in KRAS-mutant patients. In FRESCO-2, OS benefit was observed regardless of RAS mutation status, with HRs of 0.66 in RAS WT patients and 0.68 in RAS-mutant patients [50,51].

### 1.6. NEORAS Status and Potential Use of Anti-EGFR Agents

The NeoRAS phenomenon, a notable shift from RAS-mutant to wild-type (WT) status in mCRC following treatment, introduces an intriguing therapeutic avenue. This biological transformation may restore the efficacy of EGFR inhibitors previously ineffective due to RAS mutations. A multi-institutional study highlighted the occurrence of NeoRAS WT in 21.5% of 107 patients, particularly among those with non-KRAS exon 2 mutations. Clinical factors such as a smaller tumor burden and the absence of liver metastases were significantly linked to this shift, suggesting specific patient profiles may predispose individuals to NeoRAS WT emergence [52].

The SCRUM-Japan GOZILA study found 19% of patients transitioned to NeoRAS WT status, where EGFR inhibitors showed potential efficacy, achieving partial responses or stable disease for six months or more [53]. In a study by Chiara Nicolazzo et al., 43 of 72 patients (60%) with initial RAS mutations achieved WT status over 3–12 months, while 29 patients (40%) retained their RAS-mutant profile, independent of mutation subtype. Importantly, patients treated with chemotherapy in combination with bevacizumab exhibited a higher rate of RAS conversion compared to those receiving chemotherapy alone [54]. Bevacizumab use emerged as a significant factor in facilitating the NeoRAS WT shift, and patients achieving RAS WT status demonstrated improved median progression-free survival (mPFS) when treated with bevacizumab.

### 1.7. Efficacy of KRAS G12C Inhibitors in mCRC

Targeting KRAS, long considered a formidable challenge in drug development, has recently witnessed remarkable breakthroughs, particularly with KRAS (G12C) inhibitors like AMG510 (sotorasib) and MRTX849 (adagrasib) [2,3]. These inhibitors have shown promising outcomes in clinical trials.

The efficacy of the sotorasib–panitumumab combination therapy was evaluated in the phase 3 CodeBreaK-300 trial, following promising results from phase I/II trials [2]. This study assessed patients with mCRC who had progressed under standard chemotherapy. In the experimental arm, patients received a combination of sotorasib (960 mg or 240 mg) and panitumumab, while the control arm received either trifluridine-tipiracil or regorafenib. The median progression-free survival (mPFS) in the sotorasib 960 mg arm was 5.6 months, with a notable objective response rate (ORR) of 26%. In contrast, the 240 mg sotorasib arm achieved an mPFS of 3.9 months with a lower ORR of 6%. The control arm showed no objective responses, highlighting the significant therapeutic potential of the higher dose combination in this challenging clinical setting.

The phase I/II KRYSTAL-1 study assessed the efficacy of adagrasib, another KRAS G12C inhibitor, in patients with mCRC who had progressed under standard chemotherapy [3]. Patients were randomized to receive either adagrasib monotherapy or a combination of adagrasib and cetuximab. The objective response rate (ORR) for the monotherapy group was 19% (95% CI, 8 to 33), while the combination therapy group exhibited a significantly higher ORR of 46% (95% CI, 28 to 66). Based on these results, the National Comprehensive Cancer Network (NCCN) has recommended the combination of sotorasib–panitumumab or adagrasib–cetuximab as standard options for patients with KRAS G12C mutation who are resistant to standard chemotherapy. Data on other KRAS G12C inhibitors from early-phase clinical trials are comprehensively presented in Table 3 of this document. This table provides detailed insights into the efficacy profiles of these emerging treatments.

KRAS G12C inhibitors, particularly in combination with EGFR antibodies, represent the most advanced targeted therapies with real clinical potential in RAS-mutant mCRC and have been incorporated into NCCN guidelines for pretreated patients.

### 1.8. Alternative Therapeutic Strategies in RAS-Mutant Colorectal Cancer Beyond Direct RAS Inhibition

KRAS activation encompasses a variety of molecular steps. In KRAS mutant cancers, the mitogen-activated protein kinase (MAPK) pathway plays an active role [63]. Therefore, the direct inhibition of KRAS and the indirect inhibition of different stages of the MAPK pathway can provide anti-cancer efficacy. Additionally, targeting the Janus kinase/signal transducer and activator of transcription (JAK/STAT) and phosphatidylinositol-3 kinase (PI3K), which affect the MAPK pathway, has been explored in the treatment of colon cancer [64].

#### 1.8.1. SOS1 Inhibitors

In KRAS mutant CRC, where the KRAS protein toggles between its GDP-bound inactive and GTP-bound active states, acting as a molecular switch, SOS1 inhibition emerges as a significant therapeutic approach [65]. The direct inhibition of SOS1 effectively inhibits guanine exchange factor (GEF) activity [52]. Selective SOS1 inhibitors, such as BAY-293 and BI-3406, have demonstrated synergistic antiproliferative activity in the KRASG12C mutant cell line by inhibiting the RAS-RAF-MEK-ERK pathway [66,67]. Notably, BI-3406 is the first orally available SOS1–KRAS interaction inhibitor and has restricted the proliferation of most tumor cells by reducing KRAS-GTP levels in KRAS mutant cancers. BI 1701963, an analog of BI-3406, is being evaluated in a phase I clinical trial in combination with trametinib or as a monotherapy in patients with metastatic KRAS mutant tumors (NCT04111458).

#### 1.8.2. SHP2 Inhibitors

Src homology region 2 domain-containing phosphatase 2 (SHP2) is a receptor-free protein-tyrosine phosphatase and regulates proliferation by activating the RAS–MAPK–ERK signaling pathway. Blocking SHP2 prevents the loading of GTP into the RAS, and it is aimed to provide anticancer activity in this way [68,69]. Following positive results in preclinical studies, phase I clinical trials of several SHP inhibitors have begun recruiting patients (NCT05163028, NCT04528836, NCT05525559, and NCT05354843). In addition, it has been shown that SHP2 inhibitors effectively overcome adaptive resistance to MEK inhibitors in KRAS mutant cancers [70,71]. However, there are concerns that trametinib and SHP2 inhibitors may be toxic. On the other hand, it has also been shown that TNO155, an SHP2 inhibitor, can increase the efficacy of KRASG12C covalent inhibitors against KRASG12C mutant mCRC [72]. TNO155 is in a phase Ib/II clinical trial in combination with the KRASG12C inhibitor JDQ443 in KRASG12C mutant CRC (NCT04699188). The effectiveness of another SHP2 inhibitor, RMC-4630, as monotherapy in mCRC (NCT03634982) is being investigated in a phase I clinical trial.

#### 1.8.3. RAF-MEK-ERK Inhibitors

The MEK protein is situated downstream of RAS in the MAPK pathway. MEK inhibitors have demonstrated efficacy across various cancers. However, due to resistance acquisition, MEK inhibitors have shown limited benefit as monotherapy in KRAS mutant CRC [73,74]. MEK inhibitors can activate the WNT pathway, another driver of colorectal tumorigenesis. The activation of the WNT pathway has been cited as a reason for the ineffectiveness of MEK inhibitors in CRC [75]. Another resistance pathway is the PI3K pathway, and combining MEK inhibition with PI3K inhibition has shown antitumorigenic efficacy in vivo [76]. However, the combination of MEK and PI3K inhibitors in clinical practice has been limited in effectiveness and identified as having high toxicity [77]. In addition to the WNT and PI3K pathways, it has been shown that MEK inhibition can also activate the JAK1/2-STAT pathway. The secondary activation of other tumor pathways following MEK inhibition has resulted in the inability to demonstrate the efficacy of MEK inhibitors as monotherapy in mCRC. Therefore, the use of drugs affecting different molecular steps in addition to MEK inhibition has been considered. Early-phase clinical trials evaluating combinations such as afatinib (a pan-HER inhibitor) with selumetinib (a MEK inhibitor), dacomitinib (a panHER inhibitor) with PD-0325901 (a MEK1/2 inhibitor), and cobimetinib (a MEK inhibitor) with atezolizumab (an anti-PDL1), have failed to prove efficacy [78,79,80]. It is known that anti-EGFR monoclonal antibodies, the standard treatment in RAS wild-type CRC, are ineffective in RAS mutant CRC due to mutations in the steps below the EGFR pathway. Consequently, a phase Ib/II study combining MEK inhibitors with anti-EGFR agents was planned, but it yielded an objective response rate of 6.7% while severe toxicities were observed in 30–62.5% of cases [81].

Following these negative combination studies, the combination of MEK inhibition with CDK4/6 inhibition has emerged. The combination of CDK 4/6 inhibitors with MEK inhibition has been shown to synergistically reduce the growth of KRAS-mutant CRC both in vitro and in vivo [82]. Four clinical trials evaluating this combination have commenced, and data obtained so far suggest this approach as a promising route to indirectly target KRAS-mutant cancers (NCT03981614, NCT02022982, NCT03170206, and NCT04615312).

### 1.9. Immunotherapy

#### 1.9.1. Microsatellite Instable Colorectal Cancers

The efficacy of PD-1 inhibitors in the treatment of microsatellite instability-high (MSI-H) mCRC has been evaluated in various studies. In the phase II clinical trial Keynote-016, pembrolizumab treatment achieved a 40% objective response rate in patients diagnosed with MSI mCRC who have progression under standard treatments [83]. These results paved the way for larger studies in earlier stages. The Checkmate-142 phase II study demonstrated the effectiveness of nivolumab in previously treated MSI-H mCRC patients [84]. Upon examining the subgroup analysis of this study, it was observed that patients without RAS or BRAF mutations achieved a 41% objective response rate with nivolumab. In comparison, the rate was 27% in RAS mutant patients. In another phase II study evaluating the effectiveness of nivolumab–ipilimumab combination therapy in first-line treatment, the objective response rate with combination immunotherapy was found to be 80% in the RAS mutant patient group [85]. The effectiveness of pembrolizumab as a first-line treatment for mCRC was investigated in the phase III Keynote 177 study [86]. This study found that pembrolizumab contributed to OS and PFS across the entire patient group. In the subgroup analysis presented for PFS, pembrolizumab monotherapy showed a benefit in RAS wild-type patients (HR: 0.44 (95% CI: 0.29–0.67)), whereas no PFS benefit was demonstrated with pembrolizumab monotherapy in RAS mutant patients (HR: 1.19 (95% CI: 0.68–1.43)). The recently published Checkmate 8HW phase 3 study established the routine practice of the nivolumab–ipilimimumab combination [87,88]. In mCRC patients treated in the first line with the nivolumab–ipilimumumab combination, a PFS contribution was determined compared to standard chemotherapy or nivolumab treatment, and the efficacy of combination immunotherapy was also proven in the KRAS and NRAS mutant patient group.

When examining the outcomes of these studies, it is evident that while PD-1 inhibitors have been proven effective and are used in routine practice for MSI-H colon cancer, their efficacy appears to be more limited in patients with RAS mutations. In RAS mutant MSI-H patients, the efficacy may be enhanced through combination immunotherapies or combinations of immunotherapy with VEGF inhibitors.

#### 1.9.2. Microsatellite Stable Colorectal Cancers

In MSS mCRC, the efficacy of immunotherapies has been evaluated in various studies, and the effectiveness of immunotherapy has not been proven. Due to the ineffectiveness of immunotherapies as monotherapy in this patient group, the efficacy of combination treatments has been investigated. In the phase 2 ARETHUSA trial, the efficacy of pembrolizumab following temozolomide (TMZ) in heavily pretreated RAS-mutant CRC patients was explored [89]. Patients with O6-methylguanine-DNA-methyltransferase (MGMT)-hypermethylated were started on TMZ. Upon progression with TMZ, those with a tumor mutation burden ≥ 20 mutations per megabase were given pembrolizumab treatment as the second phase of the study. This study demonstrated that increasing the tumor mutational burden (TMB) with TMZ priming treatment and achieving disease control with pembrolizumab could be shown in a limited number of patients. In the recently published MAYA study by Morano et al., heavily pretreated mCRC patients were started on priming treatment with TMZ. After two treatment cycles, patients who did not progress were given a combination of temozolomide–nivolumab–ipilimumab [90]. With this combination treatment, an mPFS of 7.1 months (95% CI, 5.6–8.4) was observed. All patients in the ARETHUSA study were RAS mutant patients, and the majority (72%) of the patients in the MAYA study were RAS mutant patients. In the MAYA study, there was no significant difference in the objective response rates of TMZ and IO between RAS mutant and wild patients (*p* = 0.660). Both studies offer hope that priming treatment with TMZ could turn an MSS mCRC into an immune-hot condition.

Another study evaluating the efficacy of immunotherapy as a first-line treatment in MSS mCRC patients was the phase Ib/II MEDETREME trial [91]. In this study, patients received FOLFOX–Durvalumab–Tremelimumab as first-line treatment, followed by Durvalumab maintenance therapy after 6 treatment cycles. The interim results of the study showed a 6-month PFS of 62.5%, with complete responses observed in 5 patients. The interim data from this study are promising, and long-term results are awaited. It is known that oxaliplatin leads to immunogenic cell death, and combining oxaliplatin with immunotherapy was thought to enhance the benefit. A similar protocol-designed study from China is ongoing, investigating the efficacy of first-line treatment with the Sintilimab–Capecitabine–Oxaliplatin–Bevacizumab combination [92].

In addition, the AtezoTRIBE study compared the combination of atezolizumab with triplet chemotherapy in mCRC patients with MSS (13.5 vs. 11.5 months, *p* = 012). In this study, the addition of atezolizumab to standard treatment was shown to contribute to PFS. It was determined that the contribution of atezolizumab was higher, especially in the patient group with high immunoscores.

In conclusion, although the efficacy of single-agent immunotherapies in MSS mCRC has not been demonstrated, promising results have been obtained with combination or sequential therapies. Results from larger studies are expected to hold promise for the use of immunotherapies in routine oncology practice for the treatment of RAS mutant colon cancer.

### 1.10. Future Directions and Potential Research Areas

#### 1.10.1. Emerging Therapies Targeting KRASG12C Resistance: RMC-6291 and FMC-376

Recent advancements in targeting KRASG12C mutations have introduced novel strategies to overcome the resistance associated with KRASG12C(OFF) inhibitors. Among these, RMC-6291 has emerged as a promising RAS(ON) inhibitor. This compound uniquely binds to the chaperone protein cyclophilin A, forming an inhibitory complex that disrupts the downstream activation of GTP-bound KRAS. In a Phase I clinical trial, RMC-6291 demonstrated significant anti-tumoral efficacy in patients with KRASG12C-mutated solid tumors, even in cases where resistance had developed after prior KRASG12C(OFF) inhibitor treatments [93]. This highlights its potential as a next-generation therapeutic option for challenging KRASG12C-driven cancers.

Adding to this innovation, FMC-376, a dual-action inhibitor targeting both KRASG12C(ON) and KRASG12C(OFF) states, has shown notable anti-tumor activity in preclinical in vivo models [94]. Its dual mechanism positions it as a robust candidate to address the adaptive resistance mechanisms that limit the efficacy of monofunctional inhibitors.

#### 1.10.2. KRAS G12D Mutation: High Oncogenic Potential and Innovative Therapeutic Approaches in Colorectal Cancer

The KRAS G12D mutation is seen in 12% of metastatic CRC and is the most common KRAS mutation in CRC [95]. In preclinical models, KRAS G12D has been shown to exhibit higher oncogenic potential compared to other KRAS mutations. Biologically, this mutation has a low GTP hydrolysis rate, which keeps it continuously active. In addition, the Switch II pocket of KRAS G12D lacks the reactive residues required to form a strong covalent bond, making it difficult to target [96].

Therapeutic approaches to target KRAS G12D include innovative methods such as small-molecule inhibitors and proteolysis-targeting chimeras (PROTACs). MRTX1133 and RMC-9805 are currently being tested in early-phase clinical trials (NCT05737706 and NCT06040541) in CRC patients carrying the KRAS G12D mutation. MRTX1133 exhibits high binding affinity to GDP-loaded KRAS G12D (KD: ~0.2 pM, IC50: <2 nM) and is approximately 700-fold more selective than KRAS WT [97]. This inhibitor suppresses signaling pathways by preventing KRAS G12D/GTP/RAF1 complex formation and has been shown to cause tumor regression in preclinical models. In addition, MRTX1133 has been suggested to exhibit a synergistic effect with fluorouracil [98]. PROTAC technology targets and destroys mutant KRAS proteins. In preclinical models, KRAS G12D-specific PROTACs eliminated 95% of mutant KRAS, suppressed pERK, and showed tumor regression. While KRAS G12D-targeted agents are still investigational, their high selectivity and potent preclinical activity indicate significant translational promise, especially when combined with chemotherapy or immunotherapy.

#### 1.10.3. A Novel Pan-RAS Inhibitor for Targeting KRAS Mutations in Colorectal Cancer

The KRASG12C mutation is the subtype seen in a very small portion of CRC cases. A pan-RAS inhibitor targeting the entire RAS pathway was needed to be an effective treatment agent [99].

ADT-007 is a novel pan-RAS inhibitor that effectively suppresses the growth of RAS-mutant cancer cells, irrespective of the specific RAS mutation or isoform. It binds to the nucleotide-free form of RAS proteins, preventing GTP binding and subsequent RAS activation. This inhibition leads to the suppression of downstream signaling pathways, including MAPK and AKT, resulting in cell cycle arrest and apoptosis in cancer cells [100].

The efficacy of ADT-007 has been observed across various RAS-mutant cancer cells, regardless of the specific mutation or isoform. RAS wild-type (WT) cancer cells with GTP-activated RAS due to upstream mutations also exhibit sensitivity to ADT-007 [101]. In contrast, RAS WT cancer cells harboring downstream BRAF mutations and normal cells are generally insensitive to ADT-007. This selectivity enables ADT-007 to target mutant RAS in cancer cells without disrupting the function of WT RAS isoforms in normal cells, which is crucial for cell renewal and replacement in rapidly dividing tissues [100]. ADT-007 and similar pan-RAS inhibitors offer a promising therapeutic approach for CRC and other RAS-driven cancers by selectively inhibiting mutant RAS in cancer cells while preserving the essential functions of WT RAS in normal tissues.

### 1.11. Future Perspectives: Genetic Engineering, Immunotherapy, and Vaccination Strategies Targeting RAS-Mutant CRC

#### 1.11.1. The Clustered Regularly Interspaced Short Palindromic Repeats (CRISPR)

The clustered regularly interspaced short palindromic repeats (CRISPR)/Cas9 gene-editing system has emerged as a transformative technology in cancer biology, offering novel opportunities to dissect oncogenic pathways and develop mutation-specific therapeutic strategies. In RAS-mutant CRC, CRISPR has been increasingly employed in preclinical models to interrogate pathway dependencies, identify synthetic lethal interactions, and understand resistance mechanisms to targeted therapies [102]. In particular, allele-specific CRISPR approaches targeting mutant KRAS alleles (such as G12D or G12V) have shown promise in selectively disrupting oncogenic signaling while sparing the wild-type allele [103]. Such precision editing may hold future potential for durable oncogene suppression, although significant technical challenges must be addressed before clinical application. These include off-target effects, limited delivery efficiency in solid tumors, and immunogenicity related to Cas nucleases. Several strategies—such as using high-fidelity Cas variants, inducible expression systems, or RNA-guided base editing tools—are under development to mitigate these concerns [104].

Beyond therapeutic editing, CRISPR serves as a powerful research platform in CRC by enabling the generation of isogenic cell lines and organoid models [104]. These tools accelerate drug screening, biomarker discovery, and functional validation of candidate targets. Moreover, CRISPR activation (CRISPRa) and interference (CRISPRi) systems allow researchers to modulate gene expression without inducing double-strand breaks, expanding the versatility of CRISPR beyond simple knockouts [105]. Taken together, CRISPR technologies represent a powerful dual-purpose platform: (1) an indispensable tool for modeling RAS-mutant CRC and identifying novel therapeutic vulnerabilities and (2) a promising, though still experimental, avenue for next-generation precision therapies. As delivery systems improve and off-target effects are minimized, CRISPR may evolve from a preclinical asset to a viable therapeutic modality in selected patients with RAS-driven CRC.

#### 1.11.2. Adoptive Cell Therapy

Neoantigens derived from KRAS mutations are recognized by the immune system as “nonself” and can be identified by antigen-specific T cells, making them a potential target for immunotherapy. The immune system identifies these cells through specific human leukocyte antigen (HLA) T-cell receptors (TCRs). The clinical implications of this immune response have been reported in limited studies. A recent publication presented the relationship between the KRASG12D mutation and TCRs in a patient diagnosed with metastatic mCRC with lung metastasis [106]. It was found that CD8+ T cells, possessing HLA-C08:02-restricted T-cell receptors, specifically recognize the mutant KRASG12D. Following the intravenous administration of expanding TCRs in an ex vivo environment, a complete response was observed in lung lesions, resulting in prolonged PFS. Building on these results, TCRs targeting KRASG12D and KRASG12V were produced in HLAA11:01 transgenic mice, and peripheral blood lymphocytes were generated retrovirally [107]. Clinical trials to investigate the efficacy of these lymphocytes in individuals with pancreatic and CRC are ongoing (NCT03745326, NCT03190941).

In the recently published and promising AMPLIFY-201 study, the effectiveness of the cancer vaccine ELI-002 2P was demonstrated [108]. ELI-002 2P is a lymph node-targeted immunotherapy consisting of Amphiphil (Amph)-modified G12D and G12R mutant KRAS peptides and an Amph-modified CpG oligonucleotide adjuvant. The study showed 87% of patients experienced increased KRAS-specific T-cell responses and achieved biomarker clearance. Following the promising results of this study, the ELI-002 7P molecule targeting G12D, G12R, G12V, G12C, G12A, G12S, and G13D mutant KRAS peptides was developed and is currently undergoing a phase I/II study, actively recruiting patients (NCT05726864). Future trials will help determine whether ELI-002 can serve as a platform for adjuvant immunoprevention in early-stage RAS-mutant cancers.

#### 1.11.3. Anti-RAS Vaccines

There are vaccine studies that aim to neutralize the KRAS protein. Targeted molecular immunogen technology is an immunotherapy platform based on recombinant Saccharomyces cerevisiae yeast engineered to express one or more target protein antigens [109]. Molecules produced by the yeast Saccharomyces cerevisiae activate dendritic cells and produce T cell immune responses that kill target cells expressing cancer antigens in an antigen-specific CD8+ T cell-mediated manner in human models. GI-4000, produced in this way, targets KRAS mutations in codons 12 and 61 and provides a stable response rate of 23% in patients diagnosed with mCRC in the phase I study [110].

Among the current anti-RAS vaccine studies, the mRNA-5671 vaccine trials are the most exciting. mRNA-5671 is an mRNA vaccine targeting the most prevalent KRAS mutations (G12D, G12V, G13D, and G12C). In preclinical studies, this vaccine increased CD-8-associated T-cell response. A phase I trial evaluating the efficacy of this vaccine as monotherapy or in combination with pembrolizumab is ongoing (NCT03948763).

Although early-phase, CRISPR-based approaches, neoantigen vaccines, and adoptive T-cell therapies targeting KRAS mutations may redefine treatment paradigms in the future and warrant continued clinical exploration.

## 2. Future Directions and Conclusions

Targeting RAS mutations in mCRC remains one of the most pressing challenges in oncology. While significant progress has been made in the development of allele-specific inhibitors, pan-RAS blockade remains elusive, and therapeutic resistance often limits clinical benefit. Novel approaches—including targeting downstream effectors, modulating the tumor microenvironment, and utilizing synthetic lethality—represent promising alternatives. However, translating these therapies into routine practice requires addressing key challenges such as ensuring equitable access to biomarker testing, overcoming cost-related barriers, and expanding molecular profiling infrastructure across diverse clinical settings. Furthermore, the integration of emerging technologies like artificial intelligence and bioinformatics holds substantial potential to accelerate target discovery and optimize personalized therapy strategies. Future research should focus not only on expanding the therapeutic toolbox but also on improving the accessibility and clinical applicability of precision medicine in RAS-mutant mCRC.

## Figures and Tables

**Table 1:** Phase III randomized clinical studies conducted in first-line colorectal cancer and survival analysis according to RAS mutation status.

Trial	Phase	Experimental Arm	Control Arm	PFS (Median Month, HR, Log-Rank *p*)		OS (Median Month, HR, Log-Rank *p*)	
				Ras Wt	Ras Mut	Ras Wt	Ras Mut
[13] Maughan, 2011 (COIN)	III	FOLFOX or XELOX + Cetuximab	FOLFOX or XELOX	8.6 vs. 8.6 HR: 1.04 *p* = 0.60	6.9 vs. 6.5 HR: 0.94 *p* = 0.46	17.0 vs. 17.9 HR: 1.04 *p* = 0.960	13.6 vs. 14.8 HR: 1.02 *p* = 0.800
[15] Douillard, 2014 (PRIME)	III	FOLFOX4 + Panitumumab	FOLFOX4	10.0 vs. 8.6 HR: 0.80 *p* = 0.010	7.4 vs. 9.2 HR 1.27 *p* = 0.020	23.9 vs. 19.7 HR: 0.88 *p* = 0.170	15.5 vs. 19.2 HR: 1.17 *p* = 0.140
[22] Tveit, 2012 (NORDIC VII)	III	FLOX + Cetuximab	FLOX	7.9 vs. 8.7 HR: 1.07 *p* = 0.660	9.2 vs. 7.8 HR: 0.71 *p* = 0.07	20.1 vs. 22 HR: 1.14, *p* = 0.48	21.1 vs. 20.4 HR: 1.03 *p* = 0.89
[23] Van Kutsem, 2015 (CRYSTAL)	III	FOLFIRI + Cetuximab	FOLFIRI	11.4 vs. 8.4 HR: 0.56 *p* < 0.001	7.4 vs. 7.5 HR: 1.10 *p* = 0.470	28.4 vs. 20.2 HR: 0.69 *p* = 0.0024	16.4 vs. 17.7 HR:1.05 *p* = 0.640
[17] Price, 2015 (AGITG MAX)	III	Capecitabine + bevacizumab ± mitomycin	Capecitabine	8.6 vs. 6.0 HR = 0.69 *p* = 0.030	8.8 vs. 6.2 HR: 0.65 *p* = 0.007	18.9 vs. 20.6 HR = 0.99 *p* = 0.950	20.4 vs. 22.8 HR = 0.91 *p* = 0.700
[18] Hurwitz, 2009 (AVF-2107)	III	IFL + Bevacizumab	IFL	13.5 vs. 7.4 HR: 0.44 *p* < 0.0001	9.3 vs. 5.5 HR: 0.41 *p* = 0.0008	27.7 vs. 17.6 HR: 0.58 *p* = 0.040	19.9 vs. 13.6 HR 0.69 *p* = 0.260
[19] Hecht, 2008 (PACCE)	III	Ox-CT + Bev + Pan	Ox-CT + Bev	9.8 vs. 11.5 HR: 1.36	10.4 vs. 11.0 HR: 1.25	20.7 vs. 24.5 HR: 1.89	19.3 vs. 19.3 HR: 1.02
		Iri-CT + Bev + Pan	Iri-CT + Bev	10 vs. 12.5 HR: 1.50	8.3 vs. 11.9 HR: 1.19	NE vs. 19.8 HR: 1.28	17.8 vs. 20.5 HR: 2.14
[20] Cremolini, 2015 (TRIBE)	III	FOLFOXIRI + Bevacizumab	FOLFIRI + Bevacizumab	12.8 vs. 11.0 HR: 0.84 *p* = 0.770	12.0 vs. 9.5 HR: 0.78	26.8 vs. 37.1, HR 0.78, *p* = 0.66	23.9 vs. 27.3, HR 0.88
[24] Modest, 2019 (AIO KRK0110)	III	XELIRI or FOLFIRI + Bevacizumab	FP + Bevacizumab After PD XELIRI or FOLFIRI + Bevacizumab	11.8 vs. 8.0 HR 0.54 *p* < 0.001	9.3 vs. 8.1 HR 0.87 *p* = 0.340	28.5 vs. 23.5 HR 0.64 *p* = 0.02	23.2 vs. 21.3 HR 0.92 *p* = 0.62

PFS: Progression-Free Survival, OS: Overall Survival, Wt: Wild, Mut: Mutant, HR: Hazard Ratio, FOLFOX: folinic acid + fluorouracil + oxaliplatin, FLOX: folinic acid + fluorouracil + oxaliplatin,, FOLFOXIRI: folinic acid + fluorouracil + oxaliplatin + irinotecan, IFL: irinotecan + folinic acid + fluorouracil, XELIRI: capecitaibine + irinotecan, PD: progressive disease.

**Table 2:** Phase III randomized clinical studies conducted in second- or later-line colorectal cancer and survival analysis according to RAS mutation status.

Trial	Phase	Treatment Line	Experimental Arm	Control Arm	PFS (Median Month, HR, Log-Rank *p*)		OS (Median Month, HR, Log-Rank *p*)	
					Ras Wt	Ras Mut	Ras Wt	Ras Mut
[32] Bennouna 2013 (ML18147)	III	Second line	FOLFOX or XELOX + Cetuximab	FOLFOX or XELOX	8.6 vs. 8.6 HR: 1.04 *p* = 0.60	6.9 vs. 6.5 HR: 0.94 *p* = 0.46	17.0 vs. 17.9 HR: 1.04 *p* = 0.960	13.6 vs. 14.8 HR: 1.02 *p* = 0.800
[37] Tabernero 2015 (RAISE)	III	Second line	FOLFOX4 + Ramucurimab	FOLFOX4	10.0 vs. 8.6 HR: 0.80 *p* = 0.010	7.4 vs. 9.2 HR 1.27 *p* = 0.020	23.9 vs. 19.7 HR: 0.88 *p* = 0.170	15.5 vs. 19.2 HR: 1.17 *p* = 0.140
[42] Grothey 2013 (CORRECT)	II	Later line	FOLFOX4 + Cetuximab	FOLFOX4	8.3 vs. 7.2 HR: 0.56 *p* = 0.0064	5.5 vs. 8.6 HR: 1.72 *p* = 0.015	22.8 vs. 18.5 HR: 0.85 *p* = 0.390	13.4 vs. 17.5 HR: 1.29 *p* = 0.200
[43] Li 2015 (CONCUR)	III	Later line	FLOX + Cetuximab	FLOX	7.9 vs. 8.7 HR: 1.07 *p* = 0.660	9.2 vs. 7.8 HR: 0.71 *p* = 0.07	20.1 vs. 22 HR: 1.14, *p* = 0.48	21.1 vs. 20.4 HR: 1.03 *p* = 0.89
[38] Hegewisch-Becker, 2015 (AIOKRK0207)	III	Maintenance	FOLFIRI + Cetuximab	FOLFIRI	11.4 vs. 8.4 HR: 0.56 *p* < 0.001	7.4 vs. 7.5 HR: 1.10 *p* = 0.470	28.4 vs. 20.2 HR: 0.69 *p* = 0.0024	16.4 vs. 17.7 HR: 1.05 *p* = 0.640
[39] Aparacio 2018 (PRODIGE 9)	III	Maintenance	Capecitabine plus bevacizumab ± mitomycin	Capecitabine	8.6 vs. 6.0 HR = 0.69 *p* = 0.030	8.8 vs. 6.2 HR: 0.65 *p* = 0.007	18.9 vs. 20.6 HR = 0.99 *p*: 0.950	20.4 vs. 22.8 HR = 0.91 *p*: 0.700
[34] Van Cutsem 2016 (VELOUR)	III	Second line	IFL + Bevacizumab	IFL	13.5 vs. 7.4 HR: 0.44 *p* < 0.0001	9.3 vs. 5.5 HR: 0.41 *p* = 0.0008	27.7 vs. 17.6 HR: 0.58 *p* = 0.040	19.9 vs. 13.6 HR 0.69 *p* = 0.260

PFS: Progression-Free Survival, OS: Overall Survival, Wt: Wild, Mut: Mutant, HR: Hazard Ratio, FOLFOX: folinic acid + fluorouracil + oxaliplatin, FLOX: folinic acid + fluorouracil + oxaliplatin. Note: In the ML18147 trial, an interaction test indicated that the benefit of bevacizumab was independent of KRAS mutation status (*p* = 0.443 for PFS, *p* = 0.126 for OS).

**Table 3:** Clinical study results of KRASG12C-targeted agents in KRASG12C mutant tumors.

Study Name or ID	Phase	Experimental Drug(s)	ORR/DCR	mPFS	mOS *
[55] CodeBreaK 100	Phase I	Sotorasib	7.1%/73.8%	6.3 month	12.5 month
[56] CodeBreaK 100 (CRC expansion cohort)	Phase II	Sotorasib 960 mg qd	9.7%/82.3%	6.3 month	12.5 month
[57] CodeBreaK 101	Phase Ib	Sotorasib + panitumumab	30%/92.5%	8.2 month	17.9 month
[57] CodeBreaK 101 (subprotocol H)	Phase Ib	Sotorasib 960 mg qd + panitumumab + FOLFİRİ	58.1%/93.5%	8.2 month	17.9 month
[2] CodeBreaK 300	Phase III	Sotorasib 960 mg qd + panitumumab	26.4%/71.7%	5.6 month	NR
		Sotorasib 240 mg qd + panitumumab	5.7%/67.9%	3.9 month	11.9 month
		Standard of care	0%/46.3%	2.2 month	10.3 month
[58] KRYSTAL-1	Phase I/Ib	Adagrasib	50%/-	11.1 month	--
[3] KRYSTAL-1	Phase I/II	Adagrasib 600 mg bid	19%/86%	5.6 month	--
[3] KRYSTAL-1	Phase I/II	Adagrasib 600 mg bid + cetuximab	46%/100%	6.9 month	15.9 month
[59] NCT04449874	Phase Ib	Divarasib	29.1%/– 35.9%/–(400 mg)	5.6 month	--
[60] NCT04449874	Phase Ib	Divarasib 400 mg qd + cetuximab	62.5%	8 month	--
[61] NCT04585035	Phase I/II	Garsorasib 600 mg bid + cetuximab	51.7%/93.1%	7.5 month	--
[62] NCT05005234, NCT05497336	Phase Ib Phase III	Fulzerasib 600 mg bid	43.8%/87.5%	--	--

(ORR: Overall Response Rate, DCR: Disease Control Rate, PFS: Progression-Free Survival, OS: Overall Survival) * Some OS data could not be presented because they were not included in the literature.
